# Classification and characterization of nonequilibrium Higgs modes in unconventional superconductors

**DOI:** 10.1038/s41467-019-13763-5

**Published:** 2020-01-15

**Authors:** L. Schwarz, B. Fauseweh, N. Tsuji, N. Cheng, N. Bittner, H. Krull, M. Berciu, G. S. Uhrig, A. P. Schnyder, S. Kaiser, D. Manske

**Affiliations:** 1 Max Planck Institute for Solid State Research, 70569 Stuttgart, Germany; 2 RIKEN Center for Emergent Matter Science (CEMS), Wako, 351-0198 Japan; 3 Department of Physics and Astronomy, University of British Columbia, Vancouver, V6T 1Z1 Canada; 4 Department of Physics, University of Fribourg, 1700 Fribourg, Switzerland; 5 Lehrstuhl für Theoretische Physik I, Technische Universität Dortmund, 44221 Dortmund, Germany; 6 Quantum Matter Institute, University of British Columbia, Vancouver, V6T 1Z4 Canada; 7 4th Physics Institute and Research Center SCoPE, University of Stuttgart, 70569 Stuttgart, Germany

**Keywords:** Superconducting properties and materials, Theoretical physics

## Abstract

Recent findings of new Higgs modes in unconventional superconductors require a classification and characterization of the modes allowed by nontrivial gap symmetry. Here we develop a theory for a tailored nonequilibrium quantum quench to excite all possible oscillation symmetries of a superconducting condensate. We show that both a finite momentum transfer and quench symmetry allow for an identification of the resulting Higgs oscillations. These serve as a fingerprint for the ground state gap symmetry. We provide a classification scheme of these oscillations and the quench symmetry based on group theory for the underlying lattice point group. For characterization, analytic calculations as well as full scale numeric simulations of the transient optical response resulting from an excitation by a realistic laser pulse are performed. Our classification of Higgs oscillations allows us to distinguish between different symmetries of the superconducting condensate.

## Abstract (web summary)

The lately reported Higgs modes in unconventional superconductors require a classification and characterization allowed by nontrivial symmetry of the gap and the quench pulses. Here, the authors provide a classification scheme of Higgs oscillations with their excitation processes allowing them to distinguish between different symmetries of the superconducting condensate.

## Introduction

**Fig. 1:**
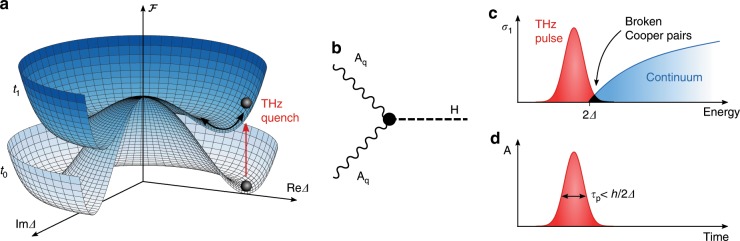
**Illustration of Higgs oscillations in a superconductor**. **a** Free energy landscape ${\mathcal{F}}$ of a superconductor as a function of the real and imaginary part of the superconducting gap $\Delta$. After a quench at ${t}_{1}> {t}_{0}$, the free energy is suddenly changed, exciting the superconducting condensate and leading to collective Higgs oscillations, indicated by a black arrow. The red arrow indicates a quench by a THz light pulse. **b** Feynman diagram describing the excitation of a Higgs mode $H$ by a light field ${\bf{A}}$ using the Raman vertex. An infrared excitation of the Higgs mode (not considered here) is only possible if an external current is present. **c** Higgs excitation mechanism using a THz quench pulse. The quench pulse only slightly overlaps with the quasi-particle continuum indicated in blue. The Mexican hat shrinks due to the breaking of Cooper pairs. **d** To excite the Higgs oscillation, the pulse must fulfill the nonadiabaticity condition in time domain.

The Higgs mode in superconductors is a collective oscillation of the order parameter $\Delta$ with the characteristic frequency of $2\Delta$. It can be understood as a massive excitation along the radial direction in the Mexican hat potential of the free energy (see Fig. 1a)^1–5^. The charge neutral Higgs mode does not couple to linear optical probes and therefore was expected to be observable only in materials with competing orders^6,7^, for which it was measured in Raman experiments^8–10^. However, an impulsive excitation of Higgs oscillations in nonequilibrium is possible via a nonlinear process by quenching the Mexican hat potential with an ultrafast THz light pulse. Such a quantum quench was demonstrated for the first time in the $s$-wave superconductor Nb${}_{1-x}$Ti${}_{x}$N^11–14^.

There are several works indicating that the spectrum of Higgs modes can be more complex if nontrivial gap symmetries are involved. Studies on multiband superconductors like MgB${}_{2}$ show that the Higgs oscillation spectrum contains Higgs modes for both gaps as well as the Leggett mode, the relative phase mode^15,16^. Additional Higgs modes with lower energies, representing oscillations of the gap in different symmetry channels, were proposed for $d$-wave superconductors under the assumption of a composite pairing interaction^17^. Besides first quench-probe experiments on cuprates^18–20^, a recent experiment on several types of cuprates shows clear fingerprints of a $2\Delta$ Higgs mode and a so far unknown additional mode below $2\Delta$^21^. These findings require both a deeper understanding and a classification and characterization of Higgs modes in nonequilibrium.

So far all descriptions on how to excite Higgs oscillations with a quench pulse are working within the dipole approximation, i.e. neglecting the small wave momentum ${\bf{q}}$ of the external field. There are other studies which show that a linear coupling of the vector potential to the condensate is possible if momentum transfer is involved, either by impurity scattering in dirty superconductors^22–26^ or in current-carrying states^27,28^.

Independent of the actual coupling to the external field, the following instructive picture can be drawn to understand the excitation process by an ultrashort THz pulse, where Higgs oscillations are excited by taking the superconductor out of equilibrium^15,29–39^. Hereby, Cooper pairs are partially broken and the landscape of the free energy changes suddenly, i.e. the Mexican hat shrinks. Thus the THz laser pulse acts like a quantum quench^40–45^, reflecting the impulsive character of the light pulse. As long as this process is faster than the time scale of the condensate, given by ${\tau }_{\Delta }=h/(2\Delta )$, where $2\Delta$ is the energy gap of the superconductor, the condensate is unable to follow the minimum of free energy adiabatically^15,36^. Consequently, collective Higgs oscillations of the gap are excited, as sketched in Fig. 1a.

In order to excite Higgs oscillations, the laser pulse must fulfill two conditions. On the one hand, the pulse should only excite a small fraction of the Cooper pairs, enough to generate a significant quench of the Mexican hat, but not too many that the superconducting signatures would be screened by hot electrons. More specifically, a short optical pulse far above gap frequencies induces a strong Drude-peak in the optical conductivity, which would overlap with the weak signal of the Higgs oscillations. Instead a suitable pulse corresponds to a peak located in or close to the gap, which only slightly overlaps with the continuum of quasi-particles, as depicted in Fig. 1c. On the other hand, the pulse must fulfill the nonadiabaticity condition, which implies a short laser pulse (Fig. 1d) and hence requires the broad spectrum in energy domain (rather than a narrow band multicycle pulse tuned close to the gap). For typical gaps in the meV regime, a single cycle THz laser pulse is exactly on the brink of these two regimes^46^, allowing for an excitation of the Higgs oscillations without heating or photo-doping the system too much.

In this article, we show how in a nonequilibrium setup for superconductors with pairing interaction in a single channel, e.g. pure $d$-wave, oscillations of the condensate in other symmetry channels can lead to additional Higgs modes as well. We classify these oscillations of the condensate based on the irreducible representations of the point group of the underlying lattice. The resulting Higgs modes depend on the excitation symmetry and the ground-state symmetry. Our detailed analysis shows that a full description of the excitation process requires to go beyond the dipole approximation in a Raman-like process and to retain the wave momentum ${\bf{q}}$ (see Fig. 1b), which plays a crucial role in breaking the symmetry of the condensate and exciting non-${A}_{1{\rm{g}}}$ oscillations of the superconducting condensate. We show that nonequilibrium Higgs oscillations offer a unique way to investigate the symmetry and collective excitation spectrum of superconductors, which allows to completely characterize the nature of a superconducting condensate with a single class of experiments.

## Results

### Quantum quenches

While the nonequilibrium probe of collective excitations in conventional $s$-wave superconductors has been studied intensively^29–31,33,34,47–50^, the response for unconventional superconductors is still in its infancy. These systems often exhibit very complicated correlations^51,52^ and a variety of different mechanisms which can lead to superconductivity. If we want to examine the nonequilibrium response of the coherent condensate of such superconductors in general, we have to go back to the fundamental properties of these systems: the symmetry of the lattice.

According to group theory, every configuration of the condensate at a given time can always be decomposed with respect to the different irreducible representations of the point group symmetry of the lattice. As an example, we take a superconductor on a lattice with ${D}_{4{\rm{h}}}$ space group symmetry, which is the lattice symmetry of cuprate high-${T}_{{\rm{c}}}$ superconductors. Based on this argument, there are four different irreducible representations: ${A}_{1{\rm{g}}}$, ${A}_{2{\rm{g}}}$, ${B}_{1{\rm{g}}}$ and ${B}_{2{\rm{g}}}$. The condensate oscillations can always be decomposed into the contributions from these sectors.

**Fig. 2:**
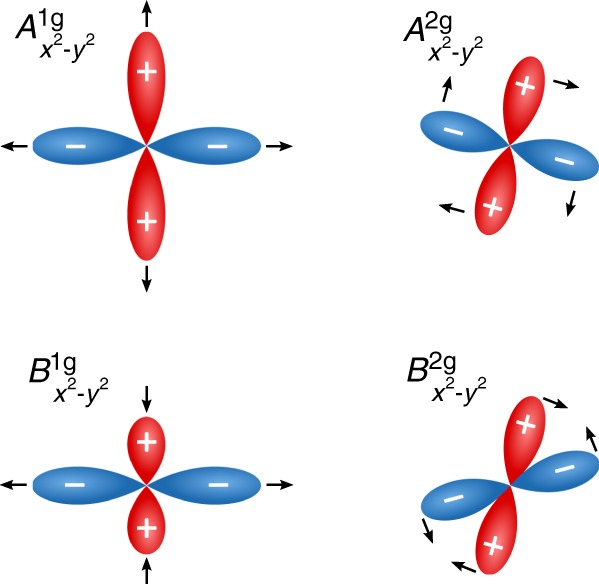
**Illustration of** $d$-**wave condensate oscillation symmetries**. Possible condensate oscillation symmetries for a ${d}_{{x}^{2}-{y}^{2}}$-wave superconductor with point group symmetry ${D}_{4{\rm{h}}}$ of the underlying lattice. The arrows indicate the motion of the lobes as a function of time. The notation of the gap symmetry in the subscript stresses the initial state, from which the oscillations of the condensate are excited.

We start our theoretical description by considering a quench of the initial state. Every quantum quench deforms the condensate from its equilibrium value, which then can be decomposed into contributions from different irreducible representations. Taking a momentum-independent quench, for example, would only probe the ${A}_{1{\rm{g}}}$ channel of the condensate^45^, but does not couple to other allowed symmetries. The solution to address also the other possible symmetries is to modify the quench and make it momentum dependent, so that we can probe other symmetry sectors as well. For example, in case of ${d}_{{x}^{2}-{y}^{2}}$-wave superconductivity, the possible oscillations of the condensate are shown in Fig. 2.

In order to illustrate this concept, we perform numerical simulations for $s$- and $d$-wave BCS superconductors to study the nonequilibrium response to momentum-dependent quantum quenches. The Hamiltonian we are investigating is given by

1 $${H}_{\text{BCS}}={H}_{0}-V\sum _{{\bf{k}},{\bf{k}}^{\prime} }\, {f}_{{\bf{k}}}{f}_{{\bf{k}}^{\prime} }{c}_{{\bf{k}}\uparrow }^{\dagger }{c}_{-{\bf{k}}\downarrow }^{\dagger }{c}_{-{\bf{k}}^{\prime} \downarrow }^{}{c}_{{\bf{k}}^{\prime} \uparrow }^{},$$

with ${f}_{{\bf{k}}}$ describing the symmetry of the interaction, $V$ the interaction strength, and the normal state Hamiltonian ${H}_{0}={\sum }_{{\bf{k}}\sigma }{\epsilon }_{{\bf{k}}}{c}_{{\bf{k}}\sigma }^{\dagger }{c}_{{\bf{k}}\sigma }$, where ${c}_{{\bf{k}}\sigma }^{\dagger }$ creates electrons with momentum ${\bf{k}}$ and spin $\sigma$. Within the BCS solution, the gap is determined by

2 $${\Delta }_{{\bf{k}}}=\Delta {f}_{{\bf{k}}}\ ,\quad \Delta =V\sum _{{\bf{k}}}\, {f}_{{\bf{k}}}\langle {c}_{-{\bf{k}}\downarrow }{c}_{{\bf{k}}\uparrow }\rangle .$$

Now we perform a quantum quench by changing the symmetry of the condensate $\langle {c}_{-{\bf{k}}\downarrow }{c}_{{\bf{k}}\uparrow }\rangle$ slightly away from its equilibrium value

3 $$\langle {c}_{-{\bf{k}}\downarrow }{c}_{{\bf{k}}\uparrow }\rangle =\frac{\Delta {f}_{{\bf{k}}}}{2{E}_{{\bf{k}}}}\quad \to \quad {\langle {c}_{-{\bf{k}}\downarrow }{c}_{{\bf{k}}\uparrow }\rangle }^{{\prime} }=\frac{\Delta {f}_{{\bf{k}}}^{{\prime} }}{2{E}_{{\bf{k}}}^{{\prime} }}$$

with ${f}_{{\bf{k}}}^{{\prime} }={f}_{{\bf{k}}}+\delta {f}_{{\bf{k}}}^{{\rm{q}}}$, where ${f}_{{\bf{k}}}^{{\rm{q}}}$ is the quench symmetry and $\delta$ the quench strength. The equilibrium value of the condensate has the symmetry of the gap ${f}_{{\bf{k}}}$ which is not changed and always remains in a single symmetry sector. After the quench, we calculate the Higgs oscillation of the order parameter as a function of time by evaluating the time-dependent gap Eq. (2), which sums the oscillations of the condensate in momentum space. For the temporal evolution, we use Anderson pseudospin formalism^53^, where the time-evolution is governed by Bloch equations. More details are given in the Methods.

For a given gap symmetry, there are different oscillations possible for the condensate, which can be excited depending on the symmetry of the quench. We use a new notation to describe this oscillation symmetry, which takes the gap symmetry into account. We add as an additional information the gap symmetry as subscript to the group theoretic notation of the irreducible representation name. We observe that depending on gap and quench symmetries not only the well-known $2\Delta$ Higgs mode occurs in the spectrum of the Higgs oscillation, which appears independent on the quench due to coupling between the modes, but also a second mode at lower energy.

**Fig. 3:**
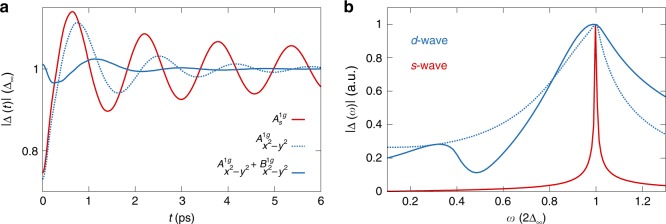
**Higgs oscillations of a** $d$-**wave superconductor**. **a** Numerical simulation of the Higgs oscillations induced by various quench symmetries for a ${d}_{{x}^{2}-{y}^{2}}$-wave superconductor. The solid (dotted) blue line shows the gap oscillations after a ${f}_{{\bf{k}}}^{{\rm{q}}} \sim 1$ (${f}_{{\bf{k}}}^{{\rm{q}}} \sim {x}^{2}-{y}^{2}$) quench as a function of time. The red solid line shows an ${f}_{{\bf{k}}}^{{\rm{q}}} \sim 1$ quench for an $s$-wave superconductor for comparison. **b** Fourier spectrum $| \Delta (\omega )| =| {\rm{FT}}| \Delta (t)| |$ of the Higgs oscillations. The oscillation for the $d$-wave gap excited by the ${f}_{{\bf{k}}}^{{\rm{q}}} \sim {x}^{2}-{y}^{2}$ quench shows a single peak, similar to the $s$-wave case. The peak position corresponds to $2{\Delta }_{\infty }$, which is two times the maximum of the gap for $t\to \infty$ after the quench. For the ${f}_{{\bf{k}}}^{{\rm{q}}} \sim 1$ quench, a second peak at low energy appears resulting from ${B}_{{x}^{2}-{y}^{2}}^{1{\rm{g}}}$ oscillations of the condensate.

Two examples for this observation are shown in Fig. 3. In Fig. 3a, we see the Higgs oscillations of the ${d}_{{x}^{2}-{y}^{2}}$ gap after a ${f}_{{\bf{k}}}^{{\rm{q}}} \sim {x}^{2}-{y}^{2}$ and ${f}_{{\bf{k}}}^{{\rm{q}}} \sim 1$ quench, which excites ${A}_{{x}^{2}-{y}^{2}}^{1{\rm{g}}}$ or ${B}_{{x}^{2}-{y}^{2}}^{1{\rm{g}}}$ oscillations of the condensate. The ${f}_{{\bf{k}}}^{{\rm{q}}} \sim 1$ quench for a $s$-wave superconductor is shown in red for comparison. We highlight that the $d$-wave oscillations decay much faster than the $s$-wave oscillations. This can be traced back to the stronger dephasing of the mode due to coupling to the gapless quasi-particles in the $d$-wave case. The final value of the gap, i.e. ${\Delta }_{\infty }$, depends on the strength of the quench, i.e. how strongly the initial states deviate from the equilibrium state^42,43^. Figure 3b shows the Fourier transform of the Higgs oscillations. The large peak at $2{\Delta }_{\infty }$ corresponds to the symmetric ${A}_{{x}^{2}-{y}^{2}}^{1{\rm{g}}}$ oscillation of the condensate.

Most importantly, a second low-energy mode is visible for the $d$-wave superconductor after the ${f}_{{\bf{k}}}^{{\rm{q}}} \sim 1$ quench. This mode does not exist for pure $s$-wave superconductors and it is also not excitable by the ${f}_{{\bf{k}}}^{{\rm{q}}} \sim {x}^{2}-{y}^{2}$ quench in the $d$-wave case, as the quench has the same symmetry as the ground-state gap. Similarly for other combinations of gap and quench symmetries, additional modes can be identified. Thus there exists a direct connection between the symmetry of the gap and the existence of low-energy Higgs modes.

To understand the nature of the second mode in more detail, we perform a linear analysis of the gap dynamics after a quantum quench. Specifically, we analytically compute the dynamics of the gap according to the expansion

4 $$\Delta (t)=\Delta (0)+\delta \Delta (t),$$

in the first order of $\delta \Delta (t)$ for different initial states. Here $\Delta (0)$ is the gap at time $t=0$ directly after the quantum quench. Transforming into Laplace space with complex frequency $s$ allows us to identify the leading contributions to the gap dynamics and leads to the expression

5 $$\delta \Delta (s)=\frac{{F}_{2}(s)}{1-{F}_{1}(s)}$$

with

6 $${F}_{2}(s)\propto {\int }_{0}^{2\pi }{\rm{d}}\varphi \frac{f(\varphi )(\Delta f^{\prime} (\varphi )-\Delta (0)f(\varphi ))}{\sqrt{{s}^{2}-4({\Delta }^{2}f^{\prime} {(\varphi )}^{2}-\Delta {(0)}^{2}f{(\varphi )}^{2})}}\ldots$$

where the dots imply additional weighting factors. For ${F}_{1}(s)$, we find the same denominator in the integrand. In these expressions, we assume that the symmetry functions primarily depend on the angle $\varphi$ between ${\bf{k}}$ and the ${{\bf{k}}}_{x}$-axis. For further details on the calculation, please see Supplementary Note 1. We can see that the spectrum of Higgs oscillations is controlled in a nontrivial way by $f{(\varphi )}^{2}-f^{\prime} {(\varphi )}^{2}$ integrated over $\varphi$ weighted by additional factors. Thus, we can trace back the second mode to the difference in the symmetry between the quench and the condensate. A second mode in the Higgs oscillation spectrum is only visible if this difference leads to a second minimum in the denominator. Particularly, this happens if there are changes of the nodal directions in the condensate symmetry compared to the equilibrium value. Due to the nonexistence of nodes in the $s$-wave case, there will be no second mode visible in the Higgs oscillations for all possible condensate oscillations.

### Realistic pulse

So far we concentrated our analysis on quantum quenches, which we classified according to the deformation symmetry from the equilibrium value. To show that these results also carry over to more realistic scenarios, we calculate the response of a ${d}_{{x}^{2}-{y}^{2}}$-wave BCS superconductor coupled to a laser field. So called pump-probe experiments have been used to study the excitation and relaxation processes of superconductors^51,54^. In a pump-probe experiment, the pump pulse excites the system, and after a delay time, the probe pulse measures various properties of the transient dynamics of the system. Varying the delay time, the temporal evolution of the system after a perturbation can be studied. As the purpose of the pump pulse in our setup is to quench the system suddenly, we call this pulse a quench pulse.

The Hamiltonian describing the quench and probe pulses is given in the methods. We use the density matrix formalism^29^ to calculate the dynamics, which is exact for the Hamiltonian in Eq. (1). We assume a short and intense THz laser pulse, which excites the condensate in an anisotropic fashion and the superconducting gap starts to oscillate. For all pulses, we fixed the pulse duration to ${\tau }_{{\rm{p}}}=0.4$ ps. With this choice, we are in the nonadiabatic regime, where a generation of collective modes is possible.

Further, we varied the direction of the light wave vector ${\bf{q}}$ to study the dependence of the optical conductivity on the quench pulse. Thus, we define the angle $\phi$ between ${\bf{q}}$ and the ${{\bf{k}}}_{x}$-axis of the superconductor. The light wave vector is small compared to the Fermi wave vector $| {\bf{q}}| \ll | {{\bf{k}}}_{{\rm{F}}}|$ such that there is no excitation of Fulde–Ferrel–Larkin–Ovchinnikov (FFLO) oscillations. However, it is large enough to break the condensate symmetry as it couples offdiagonal elements in the quasi-particle distribution (see Eq. (19)). This is possible due to the Raman-like excitation, where the photon momentum can be transferred to the condensate. By choosing the angle of the quench pulse, different oscillation symmetries can be addressed selectively (see Table 1).

**Table 1:** Classification of Higgs oscillations Possible Higgs oscillations for a lattice with *D*_4h_ point group symmetry shown for *s*, ${d}_{{x}^{2}-{y}^{2}}$, *d*_*xy*_ and ${g}_{xy({x}^{2}-{y}^{2})}$ gap functions (column one).

Gap symmetry *f*_k_	Quench symmetry ${f}_{{\bf{k}}}^{{\rm{q}}}$	Pulse direction *ϕ*	Condensate oscillation ⟨*c*_−k*↓*_*c*_k*↑*_⟩(*t*)	Higgs modes
*s*	1	–	${A}_{s}^{1{\rm{g}}}$	
	*xyx*^2^ − *y*^2^	–	${A}_{s}^{2{\rm{g}}}+{A}_{s}^{1{\rm{g}}}$	
	*x*^2^ − *y*^2^	0	${B}_{s}^{1{\rm{g}}}+{A}_{s}^{1{\rm{g}}}$	
	*xy*	*π*∕4	${B}_{s}^{2{\rm{g}}}+{A}_{s}^{1{\rm{g}}}$	
${d}_{{x}^{2}-{y}^{2}}$	*x*^2^ − *y*^2^	–	${A}_{{x}^{2}-{y}^{2}}^{1{\rm{g}}}$	
	*xy*	–	${A}_{{x}^{2}-{y}^{2}}^{2{\rm{g}}}+{A}_{{x}^{2}-{y}^{2}}^{1{\rm{g}}}$	
	1	0	${B}_{{x}^{2}-{y}^{2}}^{1{\rm{g}}}+{A}_{{x}^{2}-{y}^{2}}^{1{\rm{g}}}$	
	*xyx*^2^ − *y*^2^	*π*∕4	${B}_{{x}^{2}-{y}^{2}}^{2{\rm{g}}}+{A}_{{x}^{2}-{y}^{2}}^{1{\rm{g}}}$	
*d*_*xy*_	*xy*	–	${A}_{xy}^{1{\rm{g}}}$	
	*x*^2^ − *y*^2^	–	${A}_{xy}^{2{\rm{g}}}+{A}_{xy}^{1{\rm{g}}}$	
	*xyx*^2^ − *y*^2^	0	${B}_{xy}^{1{\rm{g}}}+{A}_{xy}^{1{\rm{g}}}$	
	1	*π*∕4	${B}_{xy}^{2{\rm{g}}}+{A}_{xy}^{1{\rm{g}}}$	
${g}_{xy({x}^{2}-{y}^{2})}$	*xyx*^2^ − *y*^2^	–	${A}_{xy({x}^{2}-{y}^{2})}^{1{\rm{g}}}$	
	1	–	${A}_{xy({x}^{2}-{y}^{2})}^{2{\rm{g}}}+{A}_{xy({x}^{2}-{y}^{2})}^{1{\rm{g}}}$	
	*xy*	0	${B}_{xy({x}^{2}-{y}^{2})}^{1{\rm{g}}}+{A}_{xy({x}^{2}-{y}^{2})}^{1{\rm{g}}}$	
	*x*^2^ − *y*^2^	*π*∕4	${B}_{xy({x}^{2}-{y}^{2})}^{2{\rm{g}}}+{A}_{xy({x}^{2}-{y}^{2})}^{1{\rm{g}}}$	

A quench can be applied to the condensate with a certain symmetry ${f}_{{\bf{k}}}^{{\rm{q}}}$ (column two), which disturbs the ground state condensate symmetry. These quenches can be controlled by an incident THz pulse with angle *ϕ*. Pumping at an arbitrary angle corresponds to a quench in all symmetry channels. Choosing high symmetry direction (column three) allows for a selective excitation. Such a quench excites oscillations of the condensate (column four), classified by the notation of the irreducible representations of the lattice symmetry. Oscillations of the condensate lead to amplitude oscillations of the gap and the qualitative Fourier spectrum of these Higgs oscillations is illustrated in the last column, showing the possible Higgs modes. An animation on how each quench deforms a given symmetry can be found in the Supplementary Movie 1.

We compare both methods, quantum quench and quench pulse, in the Supplementary Fig. 1. Besides the time evolution of the gap, the quench-probe optical conductivity provides an experimental fingerprint to observe Higgs oscillations as well. This was explicitly shown in case of $s$-wave symmetry, i.e. as the oscillation of the conductivity depending on the delay time^33^. Thus, we use a quench pulse to induce the Higgs oscillations and a much weaker probe pulse in the same direction to measure the optical conductivity.

**Fig. 4:**
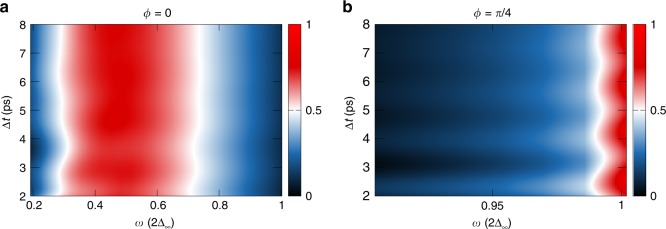
**Optical conductivity of a** $d$-**wave superconductor after excitation with a quench pulse**. Real part of the optical conductivity Re $\sigma (\Delta t,\omega )$ after a realistic quench pulse for incident angles **a** $\phi =0$ and **b** $\phi =\pi /4$ for a ${d}_{{x}^{2}-{y}^{2}}$-wave superconductor. The pulse parameters are ${\tau }_{{\rm{p}}}=0.4$ ps, $| {{\bf{A}}}_{{\rm{p}}}|$ = $7\times 1{0}^{-8}$ Js C${}^{-1}$m${}^{-1}$ and $\hslash \omega =3$ meV for the quench pulse and ${\tau }_{{\rm{p}}}=0.25$ ps, $| {{\bf{A}}}_{{\rm{p}}}|$ = $1\times 1{0}^{-8}$ Js C${}^{-1}$m${}^{-1}$ and $\hslash \omega =2.5$ meV for the probe pulse. The gap value in the simulation is $2\Delta =2.7$  meV. The vertical axis denotes the time delay between the excitation of the system with the quench pulse and the probe pulse. The horizontal axis denotes frequency $\omega$. The oscillation frequencies in $\Delta t$ correspond to the frequencies of the Higgs modes as shown in Supplementary Fig. 3.

In Fig. 4, the real part of the optical conductivity Re $\sigma (\Delta t,\omega )$ is shown for the angles $\phi =0$, along the anti-nodal direction, and $\phi =\pi /4$, along the nodal direction, as a function of frequency $\omega$ and time delay $\Delta t$. These angles correspond to the pulse direction with maximum response in Table 1.

For $\phi =\pi /4$, the symmetry breaking happens along the diagonal axis which excites in addition to the symmetric ${A}_{{x}^{2}-{y}^{2}}^{1{\rm{g}}}$ also the ${B}_{{x}^{2}-{y}^{2}}^{2{\rm{g}}}$ oscillation of the condensate and most of the weight is located at the energy of the $2\Delta$ Higgs mode. There is no low-lying peak visible in the spectrum as the ${B}_{{x}^{2}-{y}^{2}}^{2{\rm{g}}}$ oscillation does not lead to a second mode. For $\phi =0$, the ${A}_{{x}^{2}-{y}^{2}}^{1{\rm{g}}}$ and ${B}_{{x}^{2}-{y}^{2}}^{1{\rm{g}}}$ oscillations are excited, resulting in the low-energy Higgs mode and the $2\Delta$ Higgs mode and the spectrum is dominated by an in-gap response. Most importantly, the signal oscillates with respect to the time delay between quench and probe pulse, reflecting the excitation of Higgs oscillations. This is demonstrated in Supplementary Fig. 3 in more detail. Thus, the angle-resolved quench-probe experiments should be able to see Higgs oscillations in the optical conductivity, which can address different oscillation symmetries of the condensate depending on the incident angle.

### Classification of Higgs oscillations

Using the results from the quench and quench-probe calculations, an extension to other symmetries and evaluation of the Higgs oscillations enables us to write down a classification scheme for nonequilibrium Higgs modes. As an example, for the ${D}_{4{\rm{h}}}$ lattice point group, the condensate oscillations and resulting Higgs modes as well as their excitation symmetries are shown in Table 1 for all fundamental gap symmetries allowed by the point group.

For each gap symmetry ${f}_{{\bf{k}}}$ in column one, quench symmetries ${f}_{{\bf{k}}}^{{\rm{q}}}$ in all irreducible representations of the point group are listed in column two. Quenching via pump pulses at an arbitrary incident angle $\phi$ results in an excitation of all modes. Choosing a direction along the high-symmetry directions $\phi =0$ or $\phi =\pi /4$, a selective excitation of ${B}^{1{\rm{g}}}$ or ${B}^{2{\rm{g}}}$ oscillations is possible. These directions, which correspond to the respective quench symmetry with maximum intensity, are listed in the third column. As the light pulse always breaks the symmetry, it is in principle not possible to excite the symmetric ${A}^{1{\rm{g}}}$ mode alone, even in the $s$-wave case. Induced by the quench, the resulting oscillations of the condensate $\langle {c}_{-{\bf{k}}\downarrow }{c}_{{\bf{k}}\uparrow }\rangle$ are shown in column four. Independent of the quench symmetry, the symmetric ${A}^{1{\rm{g}}}$ oscillation is always excited as any disturbance leads to a global change in the quasi-particle distribution. The time-dependent amplitude of the energy gap is calculated from the gap Eq. (2), where the condensate for each momentum point is summed. This results in Higgs oscillations of the gap and a schematic picture of the spectrum is shown in the last column. For all gap symmetries, the $2\Delta$ Higgs mode is visible, which corresponds to the symmetric ${A}^{1{\rm{g}}}$ oscillation of the condensate. Depending on how the non-${A}^{1{\rm{g}}}$ oscillations change the condensate symmetry from its equilibrium symmetry, i.e. the gap symmetry, a second Higgs mode is visible in the spectrum. This is not always the case, e.g. the ${B}_{{x}^{2}-{y}^{2}}^{2{\rm{g}}}$ oscillation is not visible in the spectrum as a second Higgs mode despite its asymmetric deviation from the ground-state symmetry. As this oscillation only shifts weight inside the positive and negative lobes of the ${d}_{{x}^{2}-{y}^{2}}$ symmetry but does not move the nodal directions, it will not lead to a second mode in the summation process for the calculation of the gap oscillations. Hence, for a full analysis of the gap symmetry, information from multiple quench symmetries is required. Yet, if we can obtain this information, nonequilibrium Higgs oscillations can be used as an efficient tool to completely classify the ground-state symmetry of a superconductor.

## Discussion

To summarize, we introduce a classification scheme for nonequilibrium Higgs oscillations, which allows to characterize the ground state of superconducting condensates. Our analytical calculations show that depending on the symmetry of the quench and of the gap function, low-lying modes exist, which can be directly identified with the different oscillations of the condensate. We introduce a new notation to combine the information of the ground state with the quench symmetry in order to distinguish the different Higgs oscillations. Simulations of quench-probe experiments using realistic pulses in a microscopic model show that the usually ignored wave momentum in the dipole approximation plays an important role in the excitation of non-${A}_{1{\rm{g}}}$ oscillations of the condensate. Despite its small value compared to the Fermi wave vector, it is large enough to break the ground-state symmetry and can lead to additional Higgs modes implementing the proposed analytic quench setup.

It is important to note that the proposed experimental excitation of the Higgs mode is a Raman-like excitation and should not be confused with an infrared-active excitation^28^. In the latter case, a driven ac current would occur and thus the strength and polarization of the electric field is more important than the small momentum of the photon. In the former case, the polarization of the electric field plays a minor role and the photon momentum becomes much more important.

We find that the Higgs modes are visible in the optical conductivity of the proposed quench-probe experiment, paving the way for investigations and classifications of the dynamics of known and unknown superconductors directly within this framework. This analysis is applicable for all superconductors and requires only the knowledge of the symmetry of the crystal. It is a natural extension of the group theoretical notation to the case of nonequilibrium excitation of the system. To demonstrate the approach, we fully characterize all possible Higgs oscillations for the important ${D}_{4{\rm{h}}}$ point group, relevant, for example, for high-temperature cuprate superconductors. This main result is summarized in Table 1, which goes beyond a simple product table of gap symmetry and light pulse direction. The number of excited fundamental condensate oscillations does not directly correlate with the number of observed Higgs modes, which depend in a nontrivial way on the phase and nodal structure of the order parameter.

The experimental realization of the proposed momentum transfer to break the condensate symmetry will be challenging. If light couples to the Higgs mode only indirectly via electrons, momentum scattering on timescales faster than the oscillation period might wash out or destroy the preferred direction of the pulse. Depending on the strength of this momentum distribution effect, the second Higgs mode could be damped, might no longer follow its predicted angular dependency or may be even completely suppressed. On the other hand, recent studies have shown that impurity scattering in dirty superconductors even enhances the coupling of light to the condensate and the excitation of the Higgs mode^22–26^. As no experiments exist so far which allow to measure the transferred photon momentum in detail, the field is open for further experimental and theoretical investigations. However, the current efforts to measure Higgs oscillations on different cuprates^18–21^ show already fingerprints of collective oscillations.

It is important to note that we do not introduce additional energy scales nor other degrees of freedom, such as subdominant channels: the observed oscillations and the corresponding frequencies are intrinsic to the pure $d$-wave superconductor and do not require composite pairing symmetries. Note that we assume that no competing order, such as a charge density wave, exists. Otherwise, the spectroscopic signatures of the Higgs oscillations could be modified due to the interplay between the two phases^55^. Furthermore, effects which can modify the Higgs spectrum as well are superconductors in the strongly coupled regime^37^, coupling to Leggett modes in multiband systems^15^ or collective excitations of pair states in subleading channels, i.e. an excitation of the Bardasis–Schrieffer mode^56–59^. Other details of the normal state, such as Fermi arcs, play an unimportant role after a quantum quench of the superconducting condensate, as they only modify the scattering processes of the broken Cooper pairs. This could potentially change the damping of the Higgs oscillations, but has no effect on our proposed classification scheme.

There are different possibilities how a symmetry breaking momentum transfer to the condensate is realized in an experiment. Tilting the quench pulse direction toward the superconducting plane induces a finite in-plane momentum of the photons. More controlled momentum-dependent excitations and probes are possible in a THz-four-wave mixing^60^ or transient grating^61,62^ setup. Other possibilities include momentum-dependent scattering processes as well as coupling to other finite-momentum modes. This is discussed for example for superconductors under external current^27,28^ or as a possibility for phonon-coupled amplitudon dynamics in excitonic insulators^63,64^.

The classification and characterization of Higgs oscillations open the possibility to perform spectroscopic studies on superconductors to determine the symmetry of the order parameter. Compared to other types of measurements like ARPES or interferometry experiments using Josephson junctions, which can retrieve either amplitude or phase information, spectroscopy of Higgs oscillations with phase-stable THz lasers allows to determine amplitude and phase within a single type of quench-probe experiment. In principle, our theory for Higgs spectroscopy is not limited to characterizing equilibrium condensates, but may also be used to investigate possible light-induced superconducting states in transient states of matter^65–68^. Beyond that, Higgs spectroscopy could also be extended to investigate collective excitations of non-superconducting, symmetry broken phases, such as order parameter oscillations in excitonic insulators^63,64^ or Higgs modes in antiferromagnets^69^.

## Methods

### Extended BCS model

The Hamiltonian we are investigating is given by

7 $${H}_{\text{BCS}}={H}_{0}-\sum _{{\bf{k}},{\bf{k}}^{\prime} \in {\mathcal{W}}}{V}_{{\bf{k}}{\bf{k}}^{\prime} }{c}_{{\bf{k}}\uparrow }^{\dagger }{c}_{-{\bf{k}}\downarrow }^{\dagger }{c}_{-{\bf{k}}^{\prime} \downarrow }^{}{c}_{{\bf{k}}^{\prime} \uparrow }^{}\ ,$$

8 $${H}_{0}=\sum _{{\bf{k}},\sigma }{\epsilon }_{{\bf{k}}}{c}_{{\bf{k}}\sigma }^{\dagger }{c}_{{\bf{k}}\sigma }.$$

The normal state Hamiltonian ${H}_{0}$ is taken to be a free electron gas with an effective mass $m$. The pairing interaction ${V}_{{\bf{k}}{\bf{k}}^{\prime} }=V{f}_{{\bf{k}}}{f}_{{\bf{k}}^{\prime} }$ is assumed to be separable with the interaction strength $V$. The energy dispersion ${\epsilon }_{{\bf{k}}}={\hslash }^{2}{{\bf{k}}}^{2}/(2m)-{\epsilon }_{{\rm{F}}}$ is measured relative to the Fermi level ${\epsilon }_{{\rm{F}}}$. We apply the BCS solution in order to describe the superconducting phase. The superconducting gap equation reads

9 $${\Delta }_{{\bf{k}}}=\Delta {f}_{{\bf{k}}}\ ,\quad \Delta =V \sum _{{\bf{k}}\in {\mathcal{W}}}{f}_{{\bf{k}}}\langle {c}_{-{\bf{k}}\downarrow }{c}_{{\bf{k}}\uparrow }\rangle \ .$$

The sums in Eqs. (7) and (9) are taken over the set ${\mathcal{W}}$ of all ${\bf{k}}$ vectors with $| {\epsilon }_{\bf{k}}| \le \hslash {\omega }_{\rm{c}}$, ${\omega }_{{\rm{c}}}$ being the frequency cutoff. For a phononic glue, this corresponds to the Debye frequency. The function ${f}_{{\bf{k}}}$ is the gap symmetry function, where in the case of an $s$-wave superconductor ${f}_{\bf{k}}=1$ is a constant. In general, the symmetry function can be decomposed into the basis functions of the irreducible representations of the point group of the underlying lattice. In case of the ${D}_{4{\rm{h}}}$ group, the basis functions are shown in Supplementary Table 1, where the $d$-wave symmetry belongs to the ${B}_{1{\rm{g}}}$ representation. For all of our calculations, we assume that there is only a polar angle dependency $\varphi$ on the momentum ${\bf{k}}$ in the vicinity of the Fermi energy. Therefore, we use the functions shown in the third column.

In the ground state, the expectation values for the electron and quasi-particle distribution read

10 $$\left\langle {c}_{{\bf{k}}\uparrow }^{\dagger }{c}_{{\bf{k}}\uparrow }\right\rangle =\frac{1}{2}-\frac{{\epsilon }_{{\bf{k}}}}{2{E}_{{\bf{k}}}}\ ,\quad \left\langle {c}_{-{\bf{k}}\downarrow }{c}_{{\bf{k}}\uparrow }\right\rangle =\frac{\Delta {f}_{{\bf{k}}}}{2{E}_{{\bf{k}}}}\ ,$$

where ${E}_{{\bf{k}}}=\sqrt{{\epsilon }_{{\bf{k}}}^{2}+| {\Delta }_{{\bf{k}}}{| }^{2}}$ is the quasi-particle energy.

### Anderson pseudospin description

We define the Nambu–Gorkov spinor

11 $${\Psi }_{{\bf{k}}}=\left(\begin{array}{l}{c}_{{\bf{k}}\uparrow }\\ {c}_{-{\bf{k}}\downarrow }^{\dagger }\end{array}\right)$$

and Anderson pseudospin^53^

12 $${{\boldsymbol{\sigma }}}_{{\bf{k}}}=\frac{1}{2}{\Psi }_{{\bf{k}}}^{\dagger }{\boldsymbol{\tau }}{\Psi }_{{\bf{k}}}\ ,$$

where ${\boldsymbol{\tau }}$ are the Pauli matrices. The BCS Hamiltonian takes the form

13 $${H}_{{\rm{BCS}}}=\sum _{{\bf{k}}}{{\bf{b}}}_{{\bf{k}}}{{\boldsymbol{\sigma }}}_{{\bf{k}}}$$

with

14 $${{\bf{b}}}_{{\bf{k}}}=\left(-2\Delta {f}_{{\bf{k}}},0,2{\epsilon }_{{\bf{k}}}\right)\ ,$$

where we assume a fixed phase of the gap such that $\Delta \in {\mathbb{R}}$. In equilibrium, the $y$-component of the pseudospin is zero $\langle {\sigma }_{{\bf{k}}}^{y}\rangle =0$, whereas the $x$- and $z$-component read

15 $$\langle {\sigma }_{{\bf{k}}}^{x}\rangle =\frac{\Delta {f}_{{\bf{k}}}}{2{E}_{{\bf{k}}}}\ ,\quad \langle {\sigma }_{{\bf{k}}}^{z}\rangle =-\frac{{\epsilon }_{{\bf{k}}}}{2{E}_{{\bf{k}}}}\ .$$

At $t=0$, we apply a state quench where we change the symmetry of the condensate by changing the pseudospin expectation values

16 $$\langle {\sigma }_{{\bf{k}}}^{x}\rangle (0)=\frac{\Delta {f}_{{\bf{k}}}^{{\prime} }}{2E_{\bf{k}}^{\prime} },\quad \langle {\sigma }_{{\bf{k}}}^{z}\rangle (0)=-\frac{{\epsilon }_{{\bf{k}}}}{2{E}_{{\bf{k}}}^{{\prime} }},$$

where ${E}_{{\bf{k}}}^{{\prime} }=\sqrt{{\epsilon }_{{\bf{k}}}^{2}+{(\Delta {f}_{{\bf{k}}}^{{\prime} })}^{2}}$ and ${f}_{{\bf{k}}}^{{\prime} }={f}_{{\bf{k}}}+\delta {f}_{{\bf{k}}}^{{\rm{q}}}$ with the quench symmetry ${f}_{{\bf{k}}}^{{\rm{q}}}$ and strength $\delta$. This changes the initial ground-state symmetry of the condensate, which is the same as the gap symmetry, to the quenched symmetry ${f}_{{\bf{k}}}^{{\prime} }$. Note that the gap $\Delta (0)$ at $t=0$ is different from the equilibrium gap $\Delta$ due to the sudden change of the system state. For arbitrary times, the gap equation reads

17 $$\Delta (t)=V \sum _{{\bf{k}}}{f}_{{\bf{k}}}\langle {\sigma }_{{\bf{k}}}^{x}\rangle (t)\ .$$

This renders the Hamiltonian time-dependent as the pseudomagnetic field depends on the gap. The time-evolution of the pseudospin in the quenched system is described by Bloch equations^47^

18 $${\partial }_{t}{{\boldsymbol{\sigma }}}_{{\bf{k}}}(t)={\rm{i}}\left[{H}_{{\rm{BCS}}}(t),{{\boldsymbol{\sigma }}}_{{\bf{k}}}(t)\right]={{\bf{b}}}_{{\bf{k}}}(t)\times {{\boldsymbol{\sigma }}}_{{\bf{k}}}(t)\ .$$

The Bloch Eqs. (18) can then be solved together with the time-dependent gap Eq. (17) self-consistently.

### Coupling to vector potential

The Hamiltonian describing the coupling between superconductor and quench pulse, which brings the system out of equilibrium, is modeled by

19 $${H}_{\text{Laser}}= \frac{e\hslash }{2m} \sum _{{\bf{k}},{\bf{q}},\sigma }(2{\bf{k}}+{\bf{q}}){{\bf{A}}}_{{\bf{q}}}(t){c}_{{\bf{k}}+{\bf{q}},\sigma }^{\dagger }{c}_{{\bf{k}},\sigma }\\ +\frac{{e}^{2}}{2m} \sum _{{\bf{k}},{\bf{q}},\sigma }\left({\sum }_{{{\bf{q}}}^{{\prime} }}{{\bf{A}}}_{{\bf{q}}-{{\bf{q}}}^{{\prime} }}(t){{\bf{A}}}_{{{\bf{q}}}^{{\prime} }}(t)\right){c}_{{\bf{k}}+{\bf{q}},\sigma }^{\dagger }{c}_{{\bf{k}},\sigma },$$

where ${{\bf{A}}}_{{\bf{q}}}(t)$ is the transverse vector potential^29,33^. Working within the Coulomb gauge, the quench pulse is expressed in terms of the transverse vector potential

20 $${{\bf{A}}}_{{\bf{q}}}(t)={{\bf{A}}}_{{\rm{p}}}{e}^{-{\left(\frac{2\sqrt{\mathrm{ln}2}t}{{\tau }_{{\rm{p}}}}\right)}^{2}}\left({\delta }_{{\bf{q}},{{\bf{q}}}_{p}}{e}^{-i{\omega }_{{\rm{p}}}t}+{\delta }_{{\bf{q}},-{{\bf{q}}}_{p}}{e}^{+i{\omega }_{{\rm{p}}}t}\right).$$

The quench pulse is of Gaussian shape with photon frequency ${\omega }_{{\rm{p}}}$, photon wave vector ${{\bf{q}}}_{{\rm{p}}}$, full-width at half-maximum (FWHM) ${\tau }_{{\rm{p}}}$ and amplitude ${{\bf{A}}}_{{\rm{p}}}$. For our simulations, we consider various directions of the photon wave vector ${{\bf{q}}}_{{\rm{p}}}$ and with this concomitantly various directions of the quench induced by the pulse.

### Optical conductivity

To calculate the optical conductivity, we calculate the temporal evolution of the current density as function of the time delay between the quench and probe pulse

21 $${{\bf{j}}}_{{{\bf{q}}}_{{\rm{pr}}}}(\Delta t,t)= \,\frac{-e\hslash }{2mV}\sum _{{\bf{k}},\sigma }\left(2{\bf{k}}+{{\bf{q}}}_{{\rm{pr}}}\right)\left\langle {c}_{{\bf{k}},\sigma }^{\dagger }{c}_{{\bf{k}}+{{\bf{q}}}_{{\rm{pr}}},\sigma }\right\rangle (\Delta t,t)\\ -\frac{{e}^{2}}{mV}\sum _{{\bf{k}},{\bf{q}},\sigma }{{\bf{A}}}_{{{\bf{q}}}_{{\rm{pr}}}-{\bf{q}}}\left\langle {c}_{{\bf{k}},\sigma }^{\dagger }{c}_{{\bf{k}}+{\bf{q}},\sigma }\right\rangle (\Delta t,t),$$

where $V$ is the normalization volume and ${{\bf{q}}}_{{\rm{pr}}}$ is the wave-vector of the probe pulse^29^. We neglect the second term, because it only leads to an offset of the imaginary part of the optical conductivity. Then, the optical conductivity can be calculated^33^ by computing

22 $$\sigma (\Delta t,\omega )=\frac{{j}_{{{\bf{q}}}_{{\rm{pr}}}}(\Delta t,\omega )}{i\omega {A}_{{{\bf{q}}}_{{\rm{pr}}}}(\omega )}.$$

### Density matrix formalism

In order to simulate the evolution of the system, we use methods based on an expansion of Heisenberg’s equation of motion. For the temporal evolution of the order parameter, we use the density matrix formalism^70^. The main task of this technique is to derive equations of motion for quasi-particle densities. Within this formalism, it is advantageous to perform a Bogoliubov transformation of the electron operators, which diagonalizes the Hamiltonian ${H}_{\text{BCS}}$ in the initial state. We introduce new fermionic operators ${\alpha }_{{\bf{k}}}$ and ${\beta }_{{\bf{k}}}$, with

23 $${\alpha }_{{\bf{k}}}={u}_{{\bf{k}}}{c}_{{\bf{k}}\uparrow }-{v}_{{\bf{k}}}{c}_{-{\bf{k}}\downarrow }^{\dagger },\quad {\beta }_{{\bf{k}}}^{\dagger }={v}_{{\bf{k}}}^{* }{c}_{{\bf{k}}\uparrow }+{u}_{{\bf{k}}}^{* }{c}_{-{\bf{k}}\downarrow }^{\dagger },$$

where ${u}_{{\bf{k}}}=\sqrt{(1+{\epsilon }_{{\bf{k}}}/{E}_{{\bf{k}}})/2}$ and ${v}_{{\bf{k}}}=\sqrt{(1-{\epsilon }_{{\bf{k}}}/{E}_{{\bf{k}}})/2}$. We emphasize that the coefficients ${u}_{{\bf{k}}}$ and ${v}_{{\bf{k}}}$ do not depend explicitly on time, i.e., the temporal evolution of the quasi-particle densities is computed with respect to a fixed time-independent Bogoliubov-de Gennes basis in which the initial state is diagonal.

All physical observables, such as the order parameter amplitude $| {\Delta }_{{\bf{k}}}(t)|$ can now be expressed in terms of the new Bogoliubov quasi-particle densities $\langle {\alpha }_{{\bf{k}}}^{\dagger }{\alpha }_{{\bf{k}}^{\prime} }^{}\rangle$, $\langle {\beta }_{{\bf{k}}}^{\dagger }{\beta }_{{\bf{k}}^{\prime} }^{}\rangle$, $\langle {\alpha }_{{\bf{k}}}^{\dagger }{\beta }_{{\bf{k}}^{\prime} }^{\dagger }\rangle$ and $\langle {\alpha }_{{\bf{k}}}^{}{\beta }_{{\bf{k}}^{\prime} }^{}\rangle$. Applying the density matrix formalism for these quasi-particle densities, we get a closed set of differential equations. The ensuing differential equations are then solved on a finite size grid in momentum space. More details about the implementation can be found in refs. ^29,33^.

The pulse solution for a pumping angle of $\phi =0$ is shown in Supplementary Fig. 1. It shows a stronger broadening than the analytical calculations, but is in qualitative agreement. Note that we do not expect quantitative agreement, since a quantum quench is different from a laser pulse.

### Numerical implementation

In our simulations, we use the parameters $\Delta =1.35$ meV, ${E}_{{\rm{F}}}=9470$ meV and $m=1.9\ {m}_{{\rm{e}}}$, which are motivated by the parameters for lead^29^. However, all our computations can be rescaled to any energy scale for the gap. The numerical equations are computed on a finite size grid in momentum space in two dimensions similar to refs. ^29,33^. To obtain the required accuracy to resolve the small wave momentum ${{\bf{q}}}_{{\rm{p}}}$, we restrict our grid in a small region around the Fermi energy with a cutoff of ${E}_{{\rm{c}}}=8.3$ meV. We have ensured by varying the cutoff energy that our results do not depend on the discretization range. The $x$-direction is discretized with a step size of the wave momentum ${{\bf{q}}}_{{\rm{p}}}$, which results in 1000–2000 points. This is advantageous as we can resolve directly the coupling between the offdiagonal elements like $\langle {\alpha }_{{\bf{k}}}{\beta }_{{\bf{k}}+{\bf{q}}}\rangle$. As the coupling in $y$-direction is only indirect via the energy gap and therefore much smaller, we choose between 100 and 500 points for this direction. In total, we have of the order of $1{0}^{6}$ grid points. To reduce computational effort, we consider offdiagonal elements like $\langle {\alpha }_{{\bf{k}}}{\beta }_{{\bf{k}}+n{\bf{q}}}\rangle$ only up to $n=4$ as larger offdiagonal elements only contribute in order ${\mathcal{O}}\left({\bf{A}}_{\rm{p}}^{5}\right)$.

## Supplementary information

Supplementary Information

Description of Additional Supplementary Files

Supplementary Movie 1

## Data Availability

All relevant numerical data are available from the corresponding author upon reasonable request.

## Code availability

The numerical code used to calculate the results for this work is available from the corresponding author upon reasonable request.
