# Supplementary material for: Classification and characterization of nonequilibrium Higgs modes in unconventional superconductors
Source: Nat Commun. 2020 Jan 15;11:287. doi: 10.1038/s41467-019-13763-5 (PMC6962398; doi:10.1038/s41467-019-13763-5)
Supplement: Supplementary file 1 — Supplementary Information [file 41467_2019_13763_MOESM1_ESM.pdf]

Classification and characterization of nonequilibrium Higgs modes in unconventional superconductors: Supplementary Information

L. Schwarz,[1,*] B. Fauseweh,[1,*] N. Tsuji,[2] N. Cheng,[3] N. Bittner,[1,4] H. Krull,[5] M. Berciu,[3,6] G. S. Uhrig,[5] A. P. Schnyder,[1] S. Kaiser,[1,7] and D. Manske[1,†]

[1] *Max Planck Institute for Solid State Research, 70569 Stuttgart, Germany*
[2] *RIKEN Center for Emergent Matter Science (CEMS), Wako 351-0198, Japan*
[3] *Department of Physics and Astronomy, University of British Columbia, Vancouver V6T 1Z1, Canada*
[4] *Department of Physics, University of Fribourg, 1700 Fribourg, Switzerland*
[5] *Lehrstuhl für Theoretische Physik I, Technische Universität Dortmund, 44221 Dortmund, Germany*
[6] *Quantum Matter Institute, University of British Columbia, Vancouver V6T 1Z4, Canada*
[7] *4th Physics Institute and Research Center SCoPE, University of Stuttgart, 70569 Stuttgart, Germany*

(Dated: November 22, 2019)

SUPPLEMENTARY NOTE 1: LINEAR ANALYSIS

In order to gain an analytical insight into the evolution of the nonequilibrium equations of motion, we consider small quenches $\delta \ll 1$, such that we can linearize the deviations

$$\langle \boldsymbol{\sigma}_{\mathbf{k}} \rangle (t) = \langle \boldsymbol{\sigma}_{\mathbf{k}} \rangle (0) + \langle \delta \boldsymbol{\sigma}_{\mathbf{k}} \rangle (t) \,, \tag{1}$$

$$\Delta(t) = \Delta(0) + \delta\Delta(t) \,. \tag{2}$$

The linearized Bloch equations read

$$\partial_t \langle \delta\sigma^x_{\mathbf{k}} \rangle (t) = -2\epsilon_{\mathbf{k}} \langle \delta\sigma^y_{\mathbf{k}} \rangle (t) \,, \tag{3}$$

$$\partial_t \langle \delta\sigma^y_{\mathbf{k}} \rangle (t) = 2\epsilon_{\mathbf{k}} \frac{\Delta f'_{\mathbf{k}} - \Delta(0) f_{\mathbf{k}}}{2E'_{\mathbf{k}}} + 2\epsilon_{\mathbf{k}} \langle \delta\sigma^x_{\mathbf{k}} \rangle (t) + 2\Delta(0) f_{\mathbf{k}} \langle \delta\sigma^z_{\mathbf{k}} \rangle (t) - 2\epsilon_{\mathbf{k}} \frac{f_{\mathbf{k}}}{2E'_{\mathbf{k}}} \delta\Delta(t) \,, \tag{4}$$

$$\partial_t \langle \delta\sigma^z_{\mathbf{k}} \rangle (t) = -2 f_{\mathbf{k}} \Delta(0) \langle \delta\sigma^y_{\mathbf{k}} \rangle (t) \,. \tag{5}$$

Going into Laplace space with complex frequency s, we obtain algebraic equations and can solve for the gap

$$\delta\Delta(s) = \frac{F_2(s)}{1 - F_1(s)} \,, \tag{6}$$

where

$$F_1(s) = C - V \sum_{\mathbf{k}} f^2_{\mathbf{k}} \frac{s^2 + 4\Delta(0)^2 f^2_{\mathbf{k}}}{2E'_{\mathbf{k}} \left(s^2 + 4\epsilon^2_{\mathbf{k}} + 4\Delta(0)^2 f^2_{\mathbf{k}}\right)} \,, \tag{7}$$

$$F_2(s) = \frac{\Delta(0)}{s} (C - 1) + \frac{1}{s} V \sum_{\mathbf{k}} f_{\mathbf{k}} (\Delta f'_{\mathbf{k}} - \Delta(0) f_{\mathbf{k}}) \frac{s^2 + 4\Delta(0)^2 f^2_{\mathbf{k}}}{2E'_{\mathbf{k}} (s^2 + 4\epsilon^2_{\mathbf{k}} + 4\Delta(0)^2 f^2_{\mathbf{k}})} \,. \tag{8}$$

with $C = V \sum_{\mathbf{k}} \frac{f^2_{\mathbf{k}}}{2E'_{\mathbf{k}}} \in \mathcal{O}(1)$. With $U = V D(\epsilon_{\mathrm{F}})$, where $D(\epsilon_{\mathrm{F}})$ is the density of states at the Fermi level, we replace the momentum sum with an integral over ϵ and φ

$$V \sum_{\mathbf{k}} \to U \int_{-\infty}^{\infty} \mathrm{d}\epsilon \int_0^{2\pi} \mathrm{d}\varphi \,. \tag{9}$$

---

* Both authors contributed equally to this work

† Correspondence and requests for materials should be addressed to D.M. (d.manske@fkf.mpg.de)

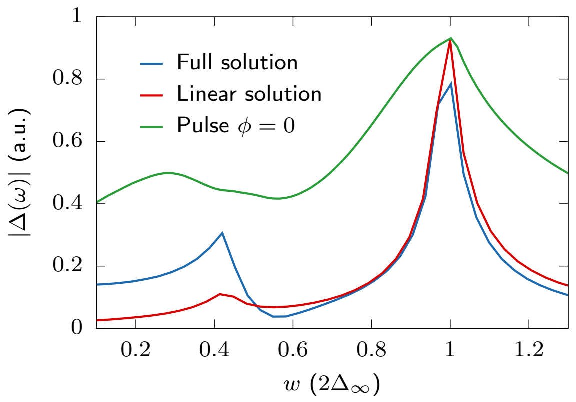

Supplementary Figure 1: Comparison between different solutions for d-wave Higgs modes Fourier spectrum $|\Delta(\omega)| = |\,\mathrm{FT}\,|\Delta(t)||$ of the gap oscillations induced by a quench $f^{\mathrm{q}}(\varphi) = 1$ and a quench pulse. The dynamics in the quenched case is calculated once in the linear approximation and once with the full solution. All three results show the 2Δ Higgs mode and a second mode below.

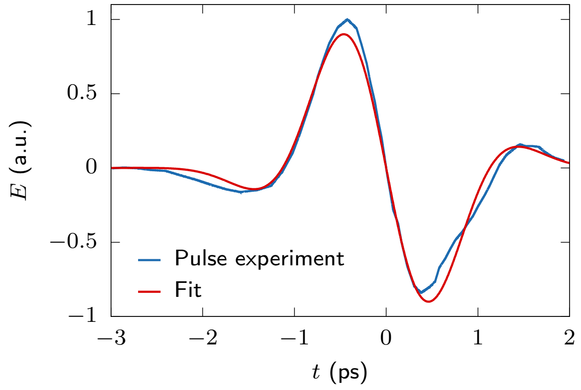

Supplementary Figure 2: Comparison between experimental pulse and theoretical parametrization. The electrical field of a single cycle THz quench pulse as used in experiment. The parameters of a fit with a Gaussian shape, as given in Equation (20) of the main text, are given by $\hbar\omega_{\mathrm{p}} = 1.33\,\mathrm{meV}$ and $\tau_{\mathrm{p}} = 1.63\,\mathrm{ps}$.

We focus first on the nominator and evaluate the ϵ integral

$$\begin{aligned} F_2(s) &= \frac{\Delta(0)}{s}(C-1) + \frac{U}{s}\int_{-\infty}^{\infty} \mathrm{d}\epsilon \int_0^{2\pi} \mathrm{d}\varphi\, \frac{f(\varphi)(\Delta f'(\varphi) - \Delta(0) f(\varphi))}{2\sqrt{\epsilon^2 + \Delta^2 f'(\varphi)^2}} \frac{s^2 + 4\Delta(0)^2 f(\varphi)^2}{s^2 + 4\epsilon^2 + 4\Delta(0)^2 f(\varphi)^2} \\ &= \frac{\Delta(0)}{s}(C-1) + \frac{U}{s}\int_0^{2\pi} \mathrm{d}\varphi \frac{f(\varphi)(\Delta f'(\varphi) - \Delta(0) f(\varphi))\sqrt{s^2 + 4\Delta(0)^2 f(\varphi)^2}}{\sqrt{s^2 - 4(\Delta^2 f'(\varphi)^2 - \Delta(0)^2 f(\varphi)^2)}} \\ &\quad \times \tanh^{-1}\left(\frac{\sqrt{s^2 - 4(\Delta^2 f'(\varphi)^2 - \Delta(0)^2 f(\varphi)^2)}}{\sqrt{s^2 + 4\Delta(0)^2 f(\varphi)^2}}\right). \end{aligned} \tag{10}$$

The expression for $F_1(s)$ reads

$$\begin{aligned} F_1(s) &= C - U\int_{-\infty}^{\infty} \mathrm{d}\epsilon \int_0^{2\pi} \mathrm{d}\varphi\, f(\varphi)^2 \frac{s^2 + 4\Delta(0)^2 f(\varphi)^2}{2\sqrt{\epsilon^2 + \Delta^2 f'(\varphi)^2}(s^2 + 4\epsilon^2 + 4\Delta(0)^2 f(\varphi)^2)} \\ &= C - U\int_0^{2\pi} \mathrm{d}\varphi\, f(\varphi)^2 \frac{\sqrt{s^2 + 4\Delta(0)^2 f(\varphi)^2}}{\sqrt{s^2 - 4(\Delta^2 f'(\varphi)^2 - \Delta(0)^2 f(\varphi)^2)}} \tanh^{-1}\left(\frac{\sqrt{s^2 - 4(\Delta^2 f'(\varphi)^2 - \Delta(0)^2 f(\varphi)^2)}}{\sqrt{s^2 + 4\Delta(0)^2 f(\varphi)^2}}\right). \end{aligned} \tag{11}$$

Without explicitly solving the integral over φ, we already see that possible modes, i.e. maxima in the function $F_2(s)$, are controlled by the difference $f(\varphi)^2 - f'(\varphi)^2$. This expression in the denominator determines the main oscillation frequencies in combination with the other weighting factors in the integrand. Depending on the gap and the quench symmetry, a low energy peak may appear as observed in the main text.

SUPPLEMENTARY NOTE 2: COMPARISON BETWEEN ANALYTICAL CALCULATIONS AND NUMERICAL RESULTS

Since we cannot solve the integral (10) analytically, we calculate the gap dynamics of the linearized equations numerically and compare it to the solution of the full equation in Supplementary Figure 1. We assume that the order parameter has d-wave symmetry, i.e. $f(\varphi) = \cos(2\varphi)$ according to Supplementary Table I, and we quench in the A_{1g} channel, i.e. $f^{\mathrm{q}}(\varphi) = 1$. Both solutions show a low energy peak, however the full nonlinear equations further amplify the intensity of the low energy mode. The energy of the lower peak depends on the quench strength, as a stronger quench increases the difference between the ground state symmetry $f(\varphi)$ and the quenched symmetry $f'(\varphi)$.

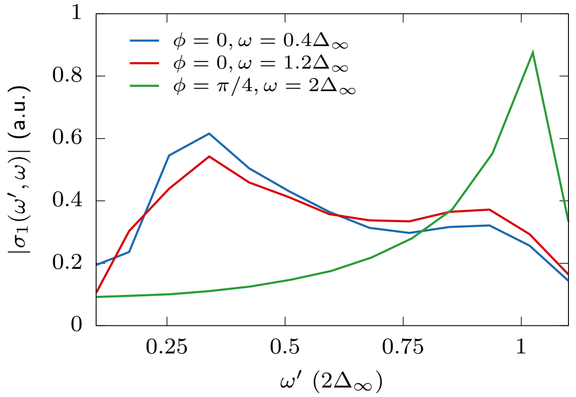

Supplementary Figure 3: Fourier transform in the delay time Δt of the optical conductivity for a *d*-wave superconductor after a quench pulse. Fourier spectrum $|\sigma_1(\omega', \omega)| = |\,\mathrm{FT}\,|\sigma_1(\Delta t, \omega)||$ of the real part of the optical conductivity for fixed frequency slices ω in Figure 4 of the main text. The red and the blue line display Higgs oscillations for a quench pulse with $\phi = 0$. The green curve corresponds to $\phi = \pi/4$.

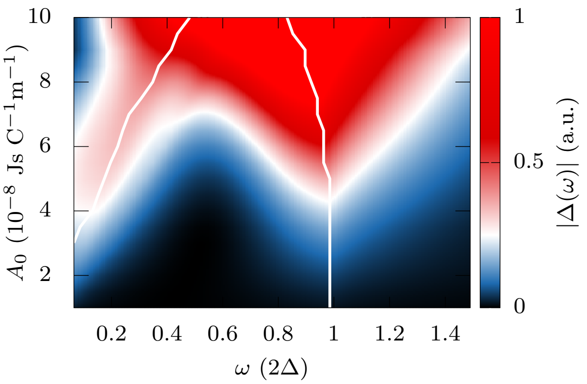

Supplementary Figure 4: Fluence dependence of the gap dynamics for $\phi = 0$ Fourier spectrum $|\Delta(\omega)| = |\,\mathrm{FT}\,|\Delta(t)||$ of the Higgs oscillations as a function of energy and laser fluence. The curves indicate the position of the 2Δ peak and of the lower-lying peak.

SUPPLEMENTARY NOTE 3: ANALYSIS OF REALISTIC QUENCH PULSE

In order to verify that our description of the experimental pulse is valid, we performed measurements of the electric field of a single cycle THz laser pulse. From the theoretical parametrization in Equation (20) of the main text we deduce the field $E(t)$ and fit it to the experimental results in Supplementary Figure 2. The fit provides an excellent approximation to the pulse used in experiment. In our simulations we rescaled the pulse width to $\tau_\mathrm{p} = 0.4$ ps and the pulse frequency to $\hbar\omega_\mathrm{p} = 3.0$ meV to adjust it to the gap parameter used.

SUPPLEMENTARY NOTE 4: FOURIER TRANSFORM OF THE OPTICAL CONDUCTIVITY

The optical conductivity shown in Figure 4 of the main text displays Higgs oscillations as function of the time delay Δt between quench and probe pulse. In Supplementary Figure 3 we analyze the Fourier spectrum of the oscillations, to verify that they correspond to the Higgs oscillations of the gap. To achieve this, we fix the frequency ω of the optical conductivity and Fourier transform the optical conductivity as function of the time delay Δt. We denote the corresponding frequency axis with ω' to distinguish it from the frequency ω. Clearly the optical conductivity shows the same peak structure as the Higgs oscillations, depending strongly on the incident angle ϕ. Thus the optical conductivity serves as an effective tool to measure Higgs oscillations.

SUPPLEMENTARY NOTE 5: FLUENCE DEPENDENCE OF THE HIGGS MODE

Besides the angular dependence of the Higgs mode in simulations with realistic quench pulses, it also depends on the intensity of the quench pulse. In Supplementary Figure 4 the Fourier transform of $\Delta(t)$ is shown for various quench pulse intensities. The 2Δ Higgs mode depends on the asymptotic value of the order parameter. With increasing intensity, more and more quasi-particles are excited and thereby the order parameter is reduced. Due to this mechanism, the frequency of the symmetric Higgs mode decreases with increasing intensity. This behavior is similar to the *s*-wave superconductor [1]. In contrast, the frequency of the second mode at lower energy is increased for higher fluence until it merges with the 2Δ Higgs mode. An increase in fluence corresponds to an increase in the quench strength. As discussed, the second mode depends strongly on the difference $f(\varphi)^2 - f'(\varphi)^2$, which increases for higher intensity of the laser. This leads to the shift to higher energy for stronger laser fluence.

Irreducible representation	Basis function	Basis function polar coordinates
$A_{1\mathrm{g}}$	1	1
$A_{2\mathrm{g}}$	$xy(x^2-y^2)$	$\sin(4\varphi)$
$B_{1\mathrm{g}}$	x^2-y^2	$\cos(2\varphi)$
$B_{2\mathrm{g}}$	xy	$\sin(2\varphi)$

Supplementary Table I: Basis functions for $D_{4\mathrm{h}}$ point group Even parity basis functions for the $D_{4\mathrm{h}}$ point group in Cartesian coordinates and as function of the azimuthal angle φ in polar coordinates [2].

SUPPLEMENTARY NOTE 6: CLASSIFICATION OF CONDENSATE OSCILLATIONS

To classify all possible condensate oscillations, we quench all fundamental gap symmetries with all fundamental quenches allowed by the $D_{4\mathrm{h}}$ point group from Supplementary Table I. In Supplementary Figure 5, we show the quenched symmetry functions for all combinations of gap and quench symmetry. After the quench, we perform a time-evolution, where we extract for each step of the time-evolution the symmetry of the condensate $\langle c_{-\mathbf{k}\downarrow}c_{\mathbf{k}\uparrow}\rangle$ for each step of the time-evolution and classify its oscillation. According to Equation (10) of the main text, the expectation value in equilibrium reads

$$\langle c_{-\mathbf{k}\downarrow}c_{\mathbf{k}\uparrow}\rangle = \frac{\Delta f_{\mathbf{k}}}{2E_{\mathbf{k}}} \,. \tag{12}$$

Using our assumption that $\epsilon_{\mathbf{k}} = \epsilon(|\mathbf{k}|)$ and $f_{\mathbf{k}} = f(\varphi)$, for k-values far away from k_{F} it follows

$$\langle c_{-\mathbf{k}\downarrow}c_{\mathbf{k}\uparrow}\rangle \approx \frac{\Delta f_{\mathbf{k}}}{2\epsilon_{\mathbf{k}}} \propto f(\varphi) \,. \tag{13}$$

We therefore use $\langle c_{-\mathbf{k}\downarrow}c_{\mathbf{k}\uparrow}\rangle$ at a k-value far from k_{F} as an approximation to trace the dynamics of the symmetry of the condensate. An animation of the condensate dynamics can be found in the Supplementary Movie 1. We name the oscillations according to its symmetry. Finally, we calculate the gap oscillation and its Fourier spectrum to obtain the collective Higgs modes resulting from the condensate oscillations.

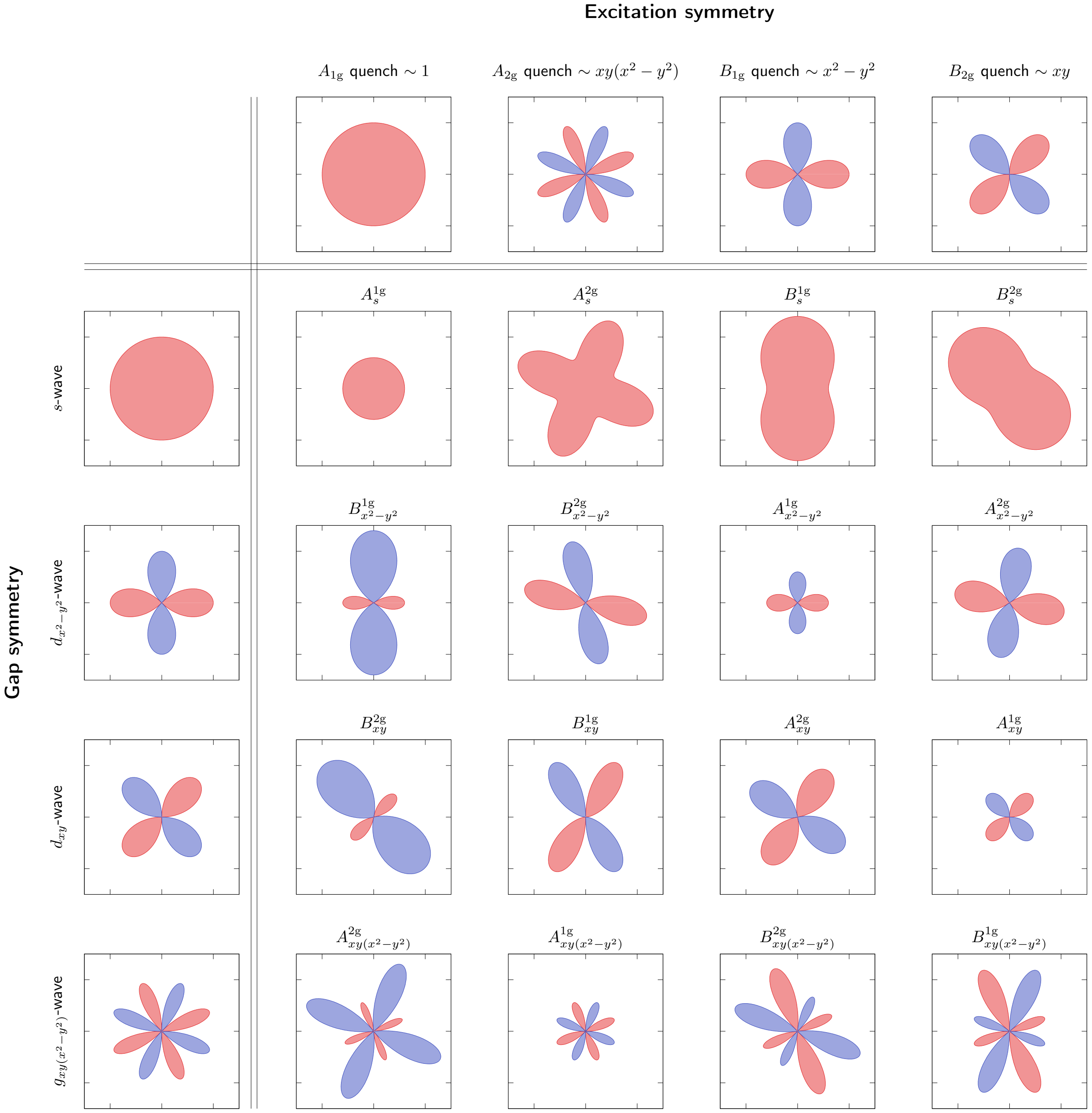

Supplementary Figure 5: Oscillation symmetries of the D_{4h} point group Oscillation symmetries of all fundamental gap symmetries allowed by the D_{4h} point group. The left column depicts the four symmetries s-, $d_{x^2-y^2}$-, d_{xy}- and $g_{xy(x^2-y^2)}$-wave i.e. the gap symmetry function $f_{\mathbf{k}}$. The top row shows possible excitation or quench symmetries $f_{\mathbf{k}}^{\mathrm{q}}$. Each cell in the graphics grid corresponds to the deformation of the gap symmetry with the respective quench symmetry, i.e. $f'_{\mathbf{k}} = f_{\mathbf{k}} + \delta f_{\mathbf{k}}^{\mathrm{q}}$ with an exemplary value of $\delta = -0.4$. The labels indicate the induced oscillations. An animation of the oscillations can be found in the Supplementary Movie 1.

[1] E. A. Yuzbashyan, O. Tsyplyatyev, and B. L. Altshuler, Relaxation and Persistent Oscillations of the Order Parameter in Fermionic Condensates, Phys. Rev. Lett. **96**, 097005 (2006).
[2] M. Sigrist and K. Ueda, Phenomenological theory of unconventional superconductivity, Rev. Mod. Phys. **63**, 239 (1991).
