# Supplementary material for: Classification and characterization of nonequilibrium Higgs modes in unconventional superconductors
Source: Nat Commun. 2020 Jan 15;11:287. doi: 10.1038/s41467-019-13763-5 (PMC6962398; doi:10.1038/s41467-019-13763-5)
Supplement: Supplementary file 2 — Description of Additional Supplementary Files [file 41467_2019_13763_MOESM2_ESM.pdf]

Description of Additional Supplementary Files

File Name: Supplementary Movie 1
Description:
Simulation of condensate dynamics for fundamental gap symmetries of the D4h point group after quenching in different symmetry channels. Each cell corresponds to the dynamics of a condensate with ground state symmetry given by the gap symmetry shown in the first column quenched with the symmetry in the first row.
