# Supplementary figures and images for: Classification and characterization of nonequilibrium Higgs modes in unconventional superconductors
Source: Nat Commun. 2020 Jan 15;11:287. doi: 10.1038/s41467-019-13763-5 (PMC6962398; doi:10.1038/s41467-019-13763-5)

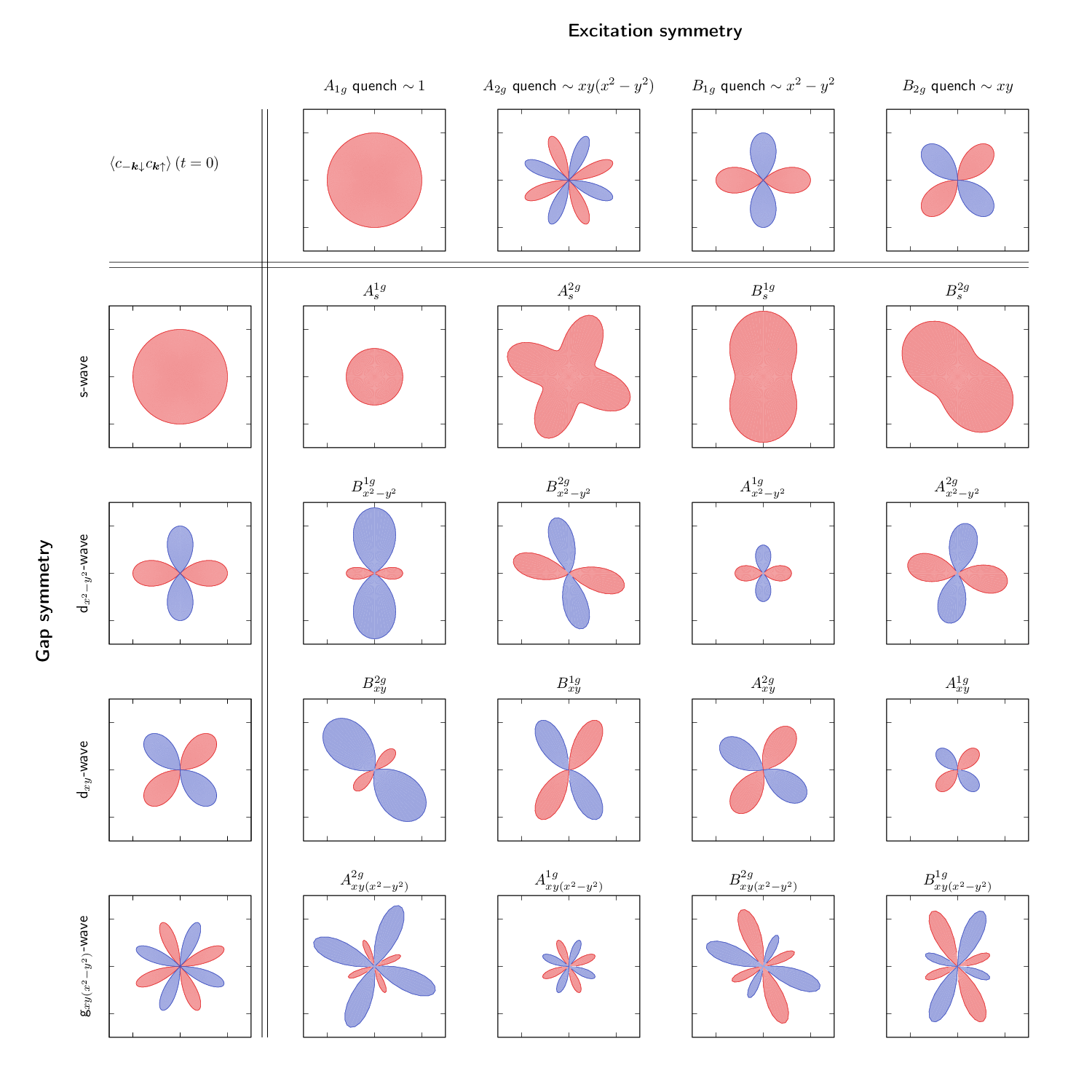

Supplement: Supplementary file 3 — Supplementary Movie 1 [file 41467_2019_13763_MOESM3_ESM.gif]
